# Supplementary material for: Single cell molecular alterations reveal target cells and pathways of concussive brain injury
Source: Nat Commun. 2018 Sep 25;9:3894. doi: 10.1038/s41467-018-06222-0 (PMC6156584; doi:10.1038/s41467-018-06222-0)
Supplement: Supplementary file 1 — Supplementary Information [file 41467_2018_6222_MOESM1_ESM.pdf]

Single Cell Molecular Alterations Reveal Target Cells and Pathways of Concussive Brain Injury

Arneson et al

Supplementary Information

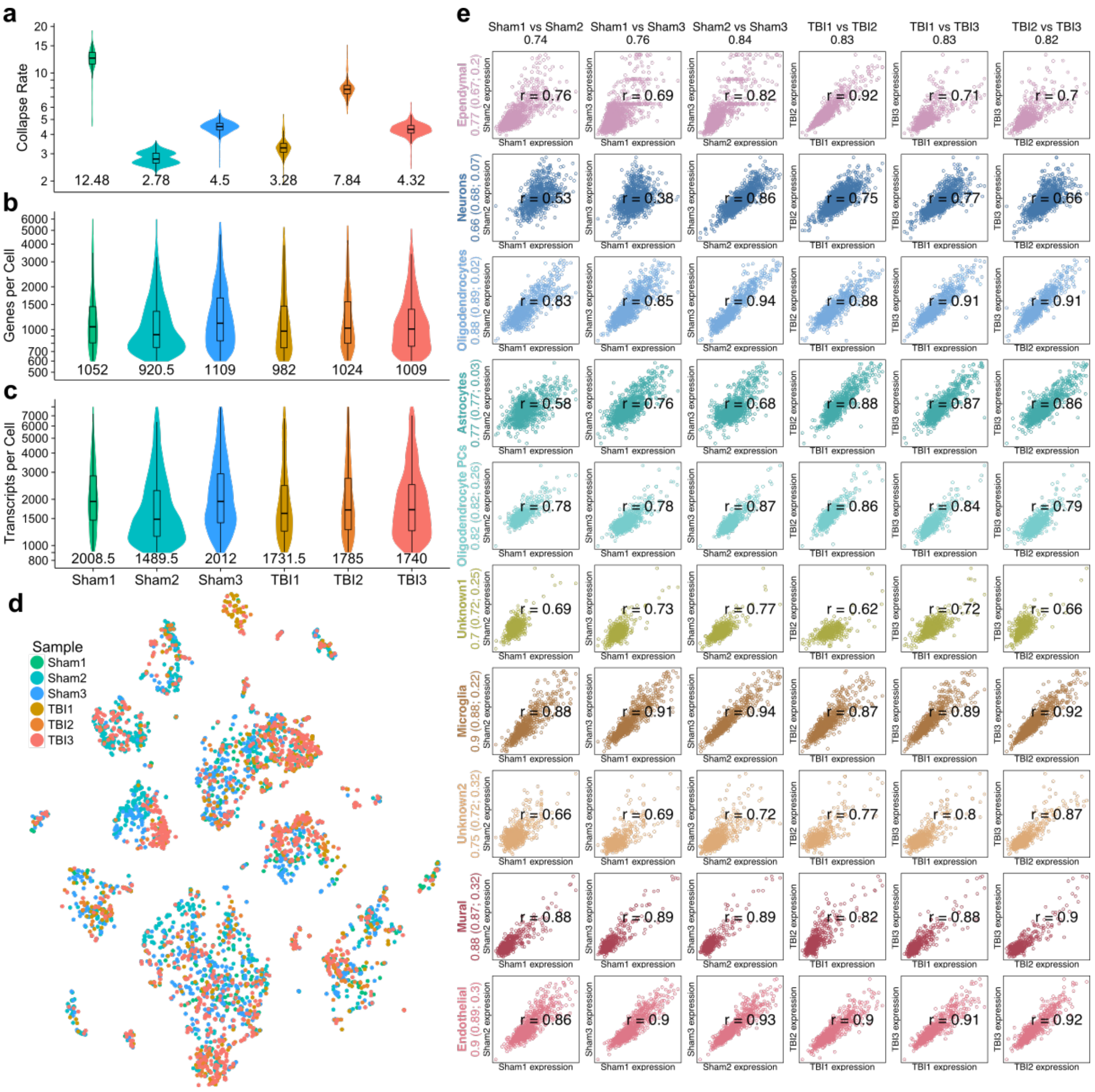

Supplementary Figure 1: Drop-seq library statistics and quality control (QC) features. **a-c).** Comparison of QC features of the 6 samples indicate that although sequencing depth varies across the samples/libraries, the number of genes and transcripts detected per cell are similar across samples. **a).** Violin/boxplot for unique molecular identifier (UMI) collapse rate for each sample, calculated as (the total number of UMIs)/(number of unique UMIs) for each single cell in a libary. UMI collapse rate is an indicator of how deeply a library is sequenced and how many reads are being discarded. **b).** Violin/boxplot for the number of unique genes detected per single cell in each sample. **c**). Violin/boxplot for the number of unique transcripts detected per single cell in each sample. **d-e).** Examination of sample batch effects on cell clusters. **d**) Single cells were colored by sample origin (3 Sham and 3 TBI animals) on the t-SNE plot. None of the clusters were driven exclusively by a sample, as single cells from all samples are present in each

cluster. We do, however, see segregation within some of the clusters due to TBI vs. Sham status, and one cluster has the majority of cells from TBI. **e**). Within- and between-group gene-gene correlations for each cell type identified indicate overall coherence of gene expression between samples. Gene-gene correlations between pairwise combinations of samples (both between and within group) were assessed for each cell type. Numbers within the individual plots indicate the correlation of gene expression levels between the two indicated samples for the specified cell type. The numbers next to the cell type labels on the left side of the plots indicate the following correlations: average within group correlations (Sham vs Sham and TBI vs TBI); average between-group correlations (Sham vs TBI); average correlation between the cell type of interest and all other cell types within the same sample. The number on the top of each column indicates the average correlation between the two indicated samples across all cell types.

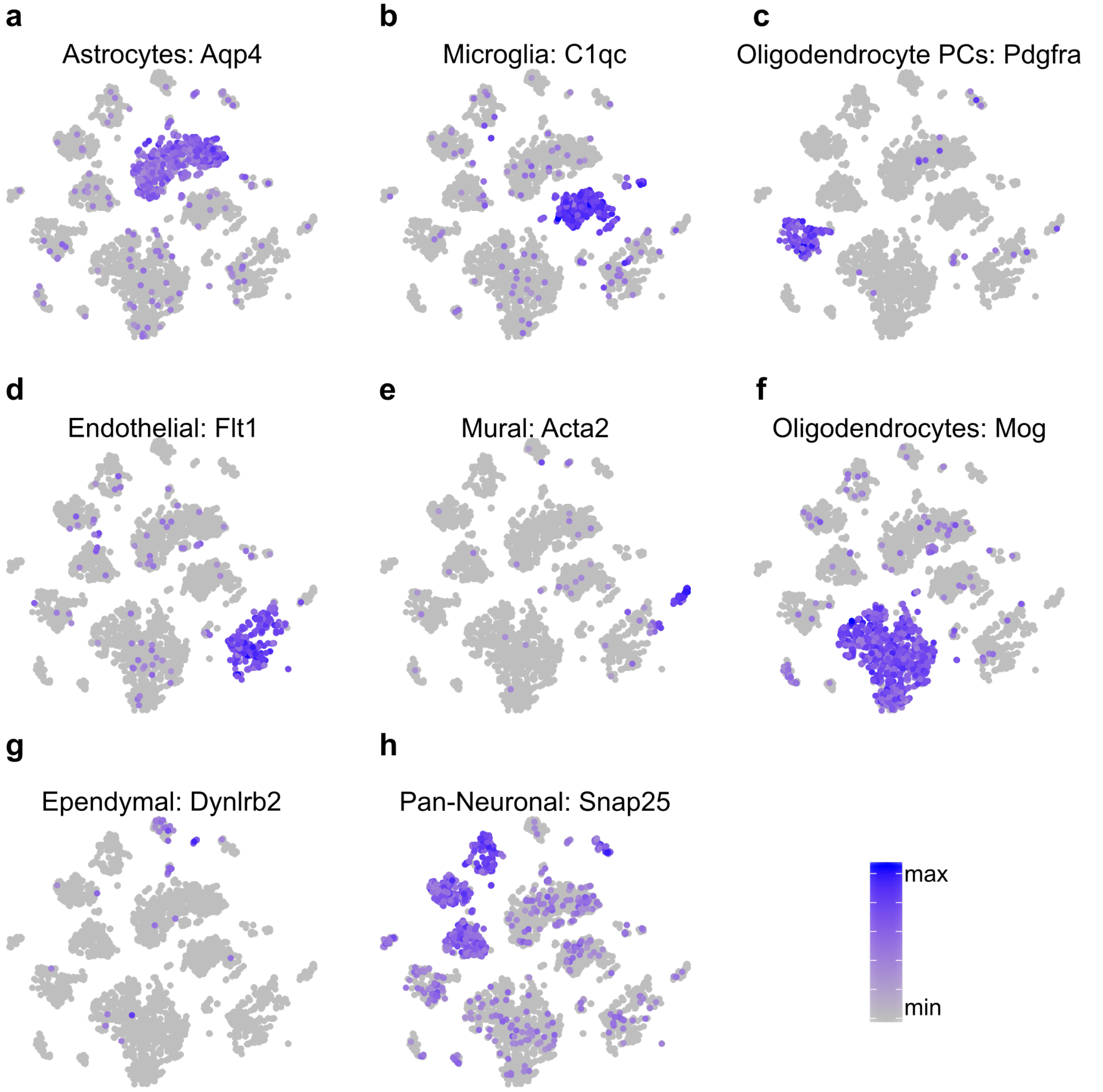

Supplementary Figure 2: Cluster specific expression of known cell-type specific markers. The expression levels of known cell-type specific markers were indicated with red color in the t-SNE plot to assign cluster identities. The following major known marker genes were used: **a).** *Aqp4* for astrocytes, **b).** *C1qc* for microglia, **c).** *Pdgfra* for oligodendrocyte progenitor cells, **d).** *Flt1* for endothelial cells, **e).** *Acta2* for mural cells, **f).** *Mog* for oligodendrocytes, **g).** *Dynlrb2* for ependymal cells, and **h).** *Snap25* for neurons.

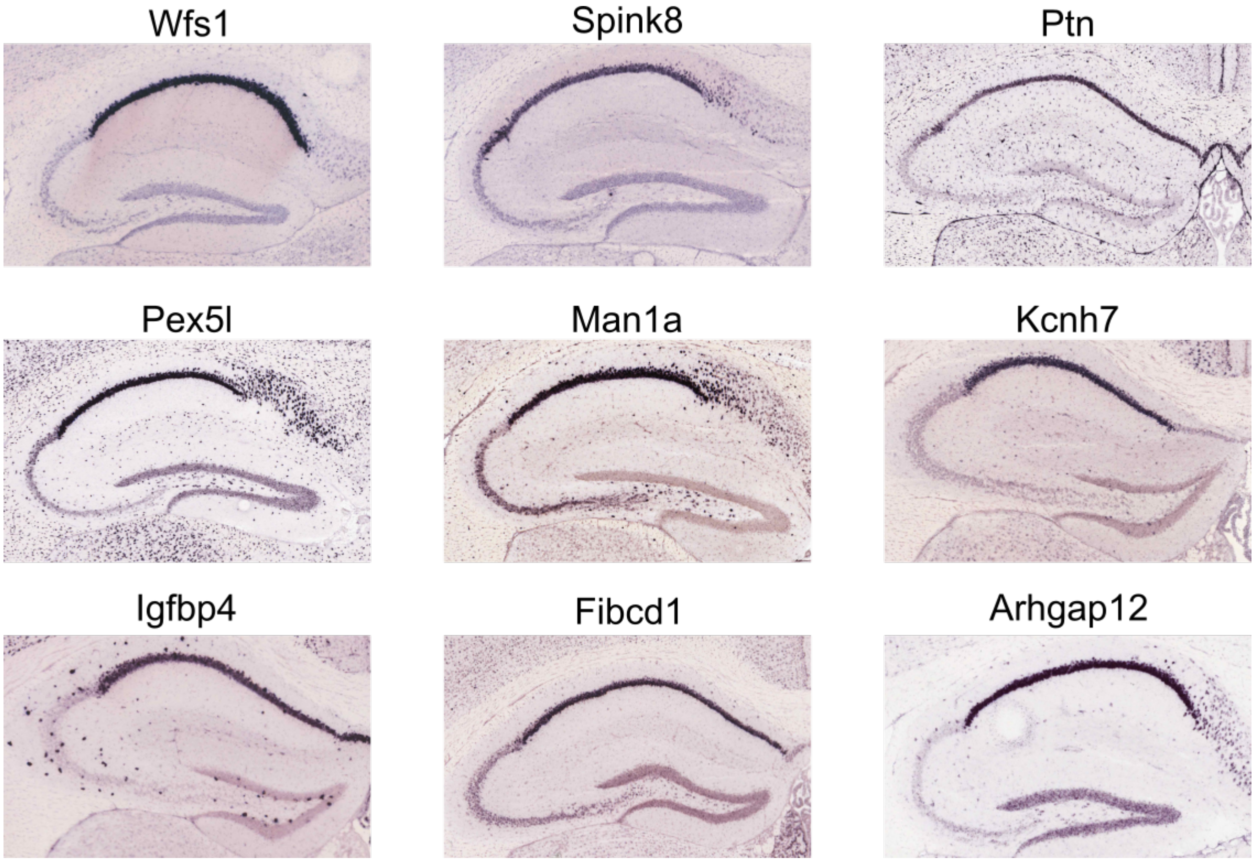

Supplementary Figure 3: CA1 neuron-specific marker gene validation with ISH images. To demonstrate the specificity of the marker genes of the CA1 neuronal cluster generated using our single cell sequencing approach we show the precise of the staining of the ISH images from the Allen Brain Atlas to the CA1 region for nine of our top marker genes of the CA1 neuronal cluster.

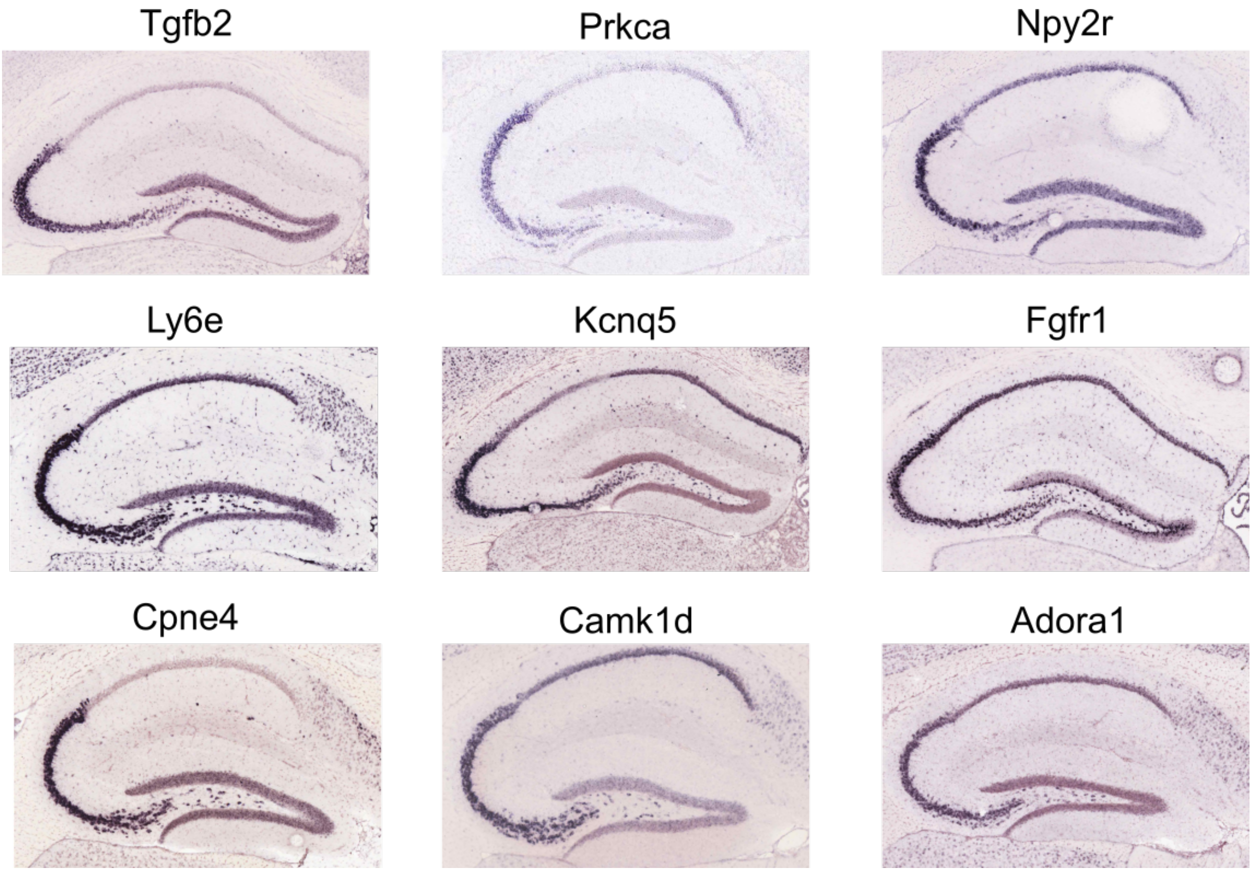

Supplementary Figure 4: CA3 neuron-specific marker gene validation with ISH images. To demonstrate the specificity of the marker genes of the CA3 neuronal cluster generated using our single cell sequencing approach we show the precise of the staining of the ISH images from the Allen Brain Atlas to the CA3 region for nine of our top marker genes of the CA3 neuronal cluster.

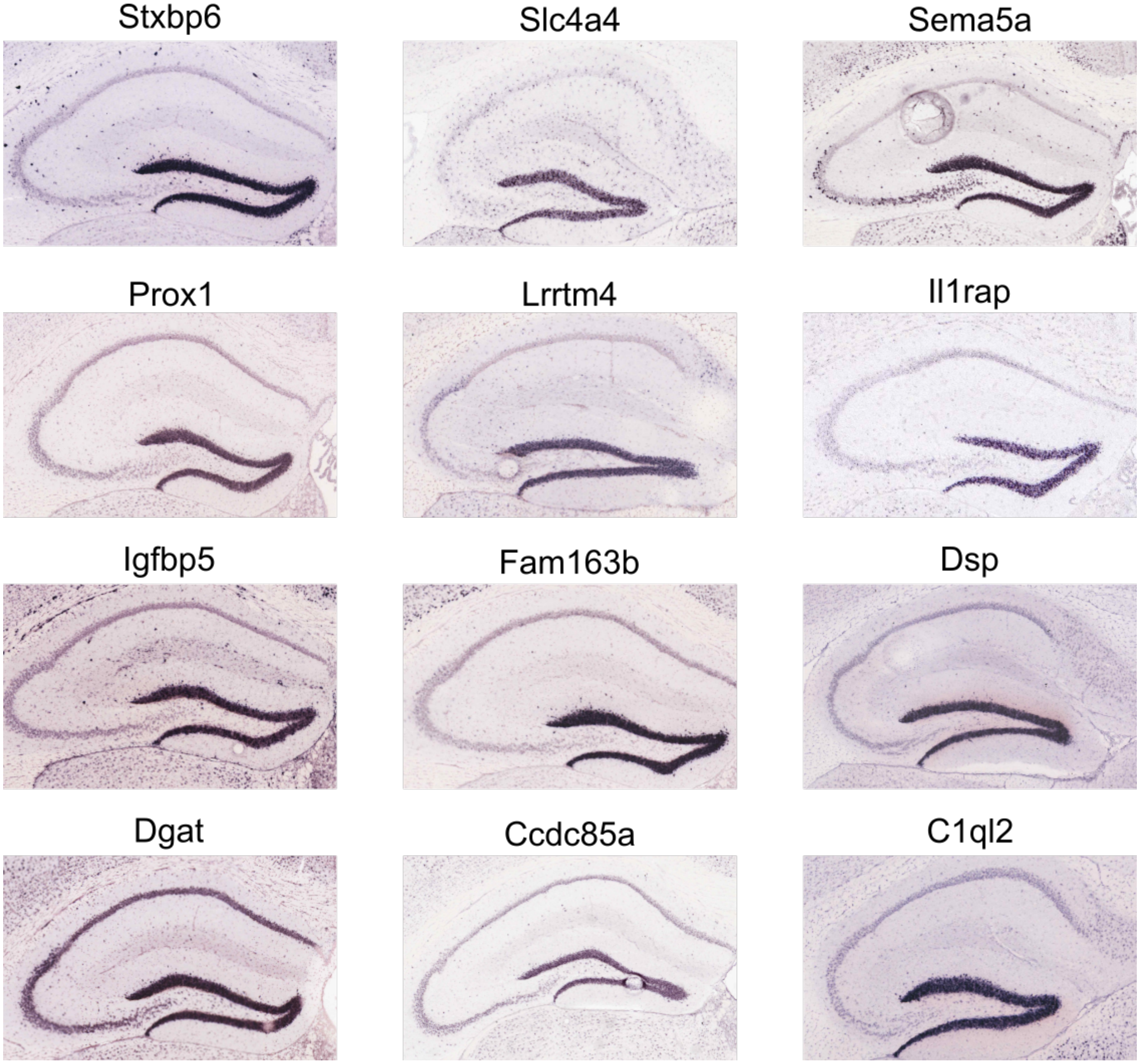

Supplementary Figure 5: DG granule cell-specific marker gene validation with ISH images. To demonstrate the specificity of the marker genes of the DG granule cell cluster generated using our single cell sequencing approach we show the precise of the staining of the ISH images from the Allen Brain Atlas to the DG region for twelve of our top marker genes of the DG granule cell cluster.

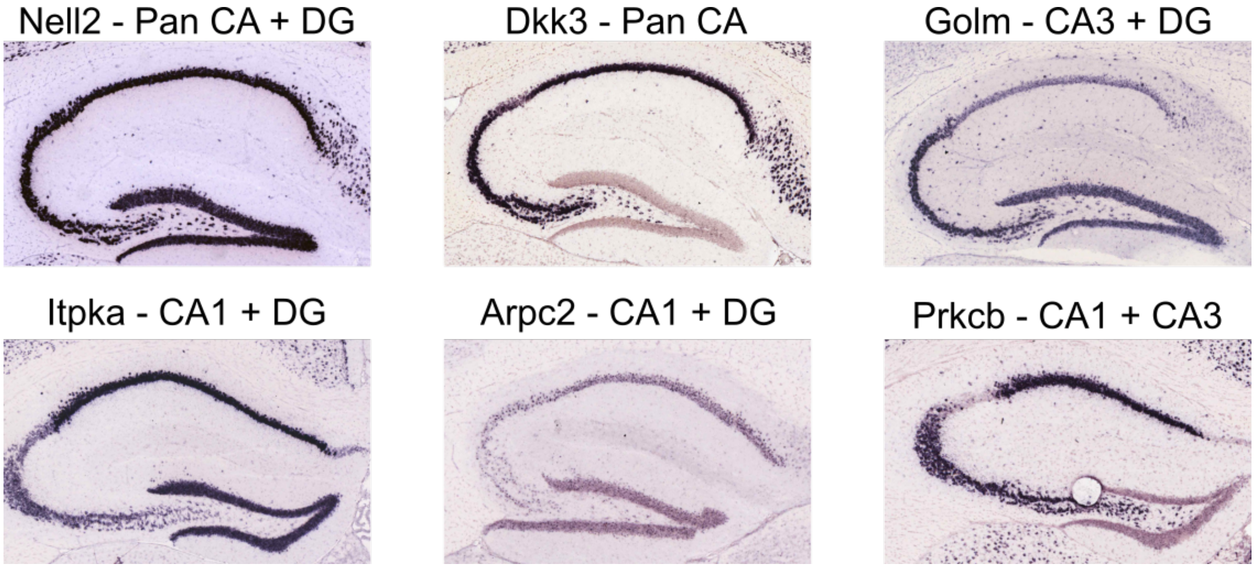

Supplementary Figure 6: Neuron cluster-specific marker gene validation with ISH images. Some of the marker genes for our neuronal clusters are preesent in more than one neuronal cell type. To demonstrate that these marker genes are indeed markers of multiple neuronal clusters we show the precise of the staining of the ISH images from the Allen Brain Atlas to the specified regions for six marker genes seen across multiple neuronal cell types.

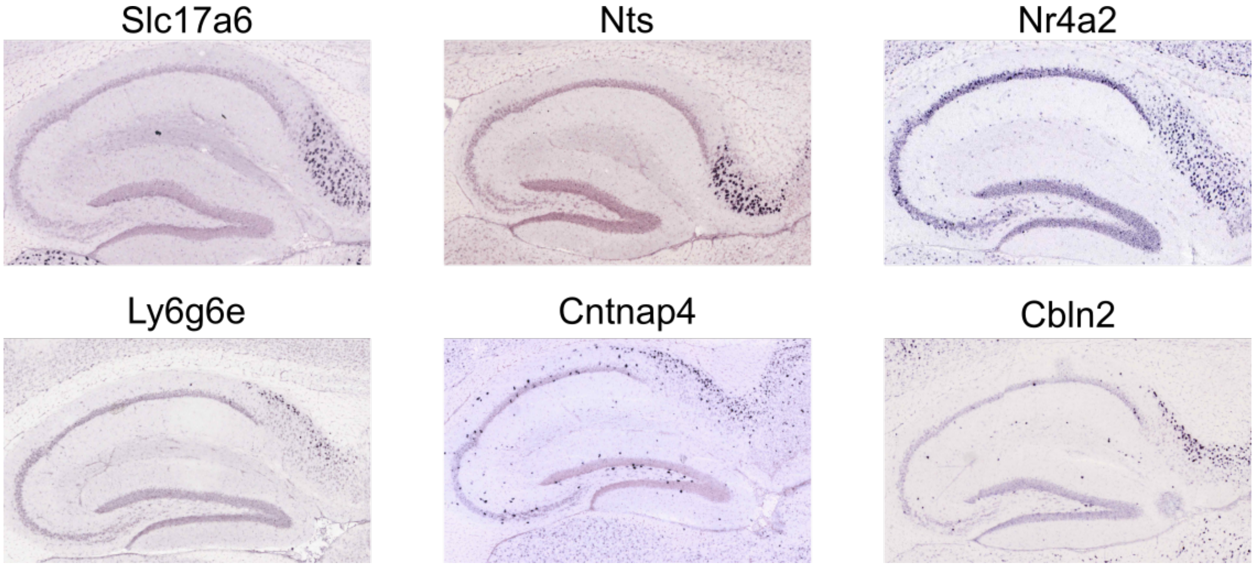

Supplementary Figure 7: CA Subtype 2 neuron cluster-specific marker gene validation. To demonstrate the specificity of the marker genes of the CA Subtype 2 neuron cluster and to help refine the cell type represented by this set of single cells, we show the precise of the staining of the ISH images from the Allen Brain Atlas to the cells inside the ventricles for nine of our top marker genes of the CA Subtype 2 neuron cluster.

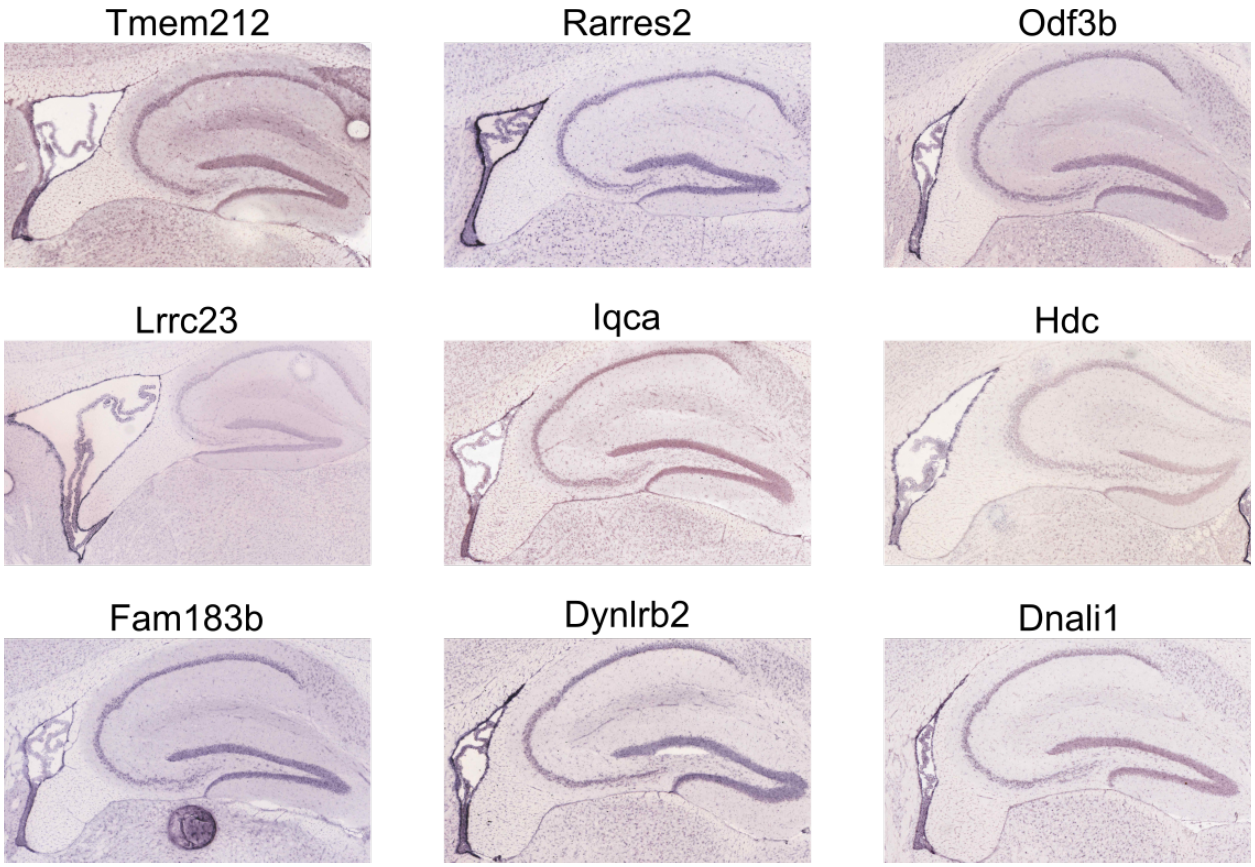

Supplementary Figure 8: Ependymal-specific marker gene validation with ISH images. To demonstrate the specificity of the marker genes of the Ependymal cluster generated using our single cell sequencing approach we show the precise of the staining of the ISH images from the Allen Brain Atlas to the Ependymal cells lining the ventricles for nine of our top marker genes of the Ependymal cluster.

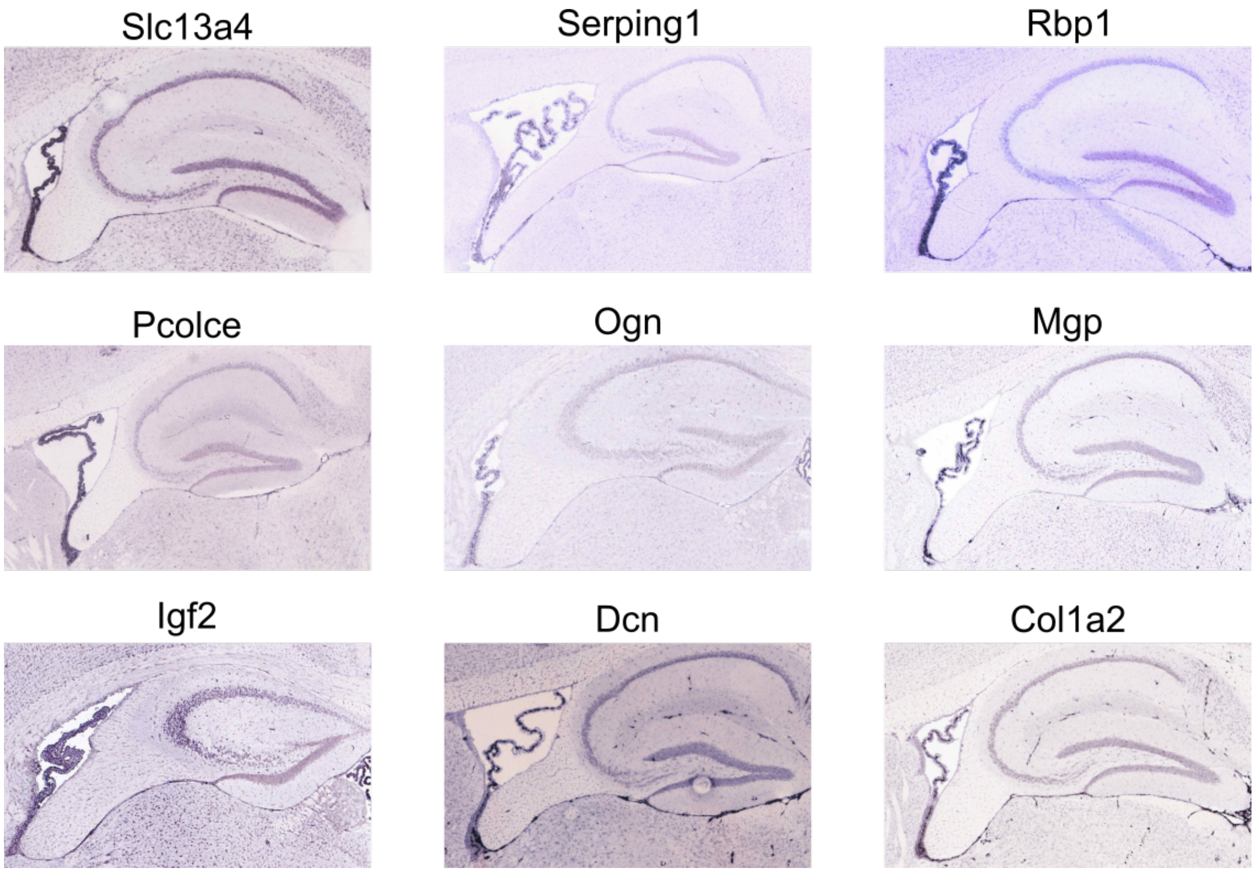

Supplementary Figure 9: Unknown2-specific marker gene validation with ISH images. To demonstrate the specificity of the marker genes of the Unknown2 cluster and to help refine the cell type represented by this set of single cells, we show the precise of the staining of the ISH images from the Allen Brain Atlas to the cells inside the ventricles for nine of our top marker genes of the Unknown2 cluster.

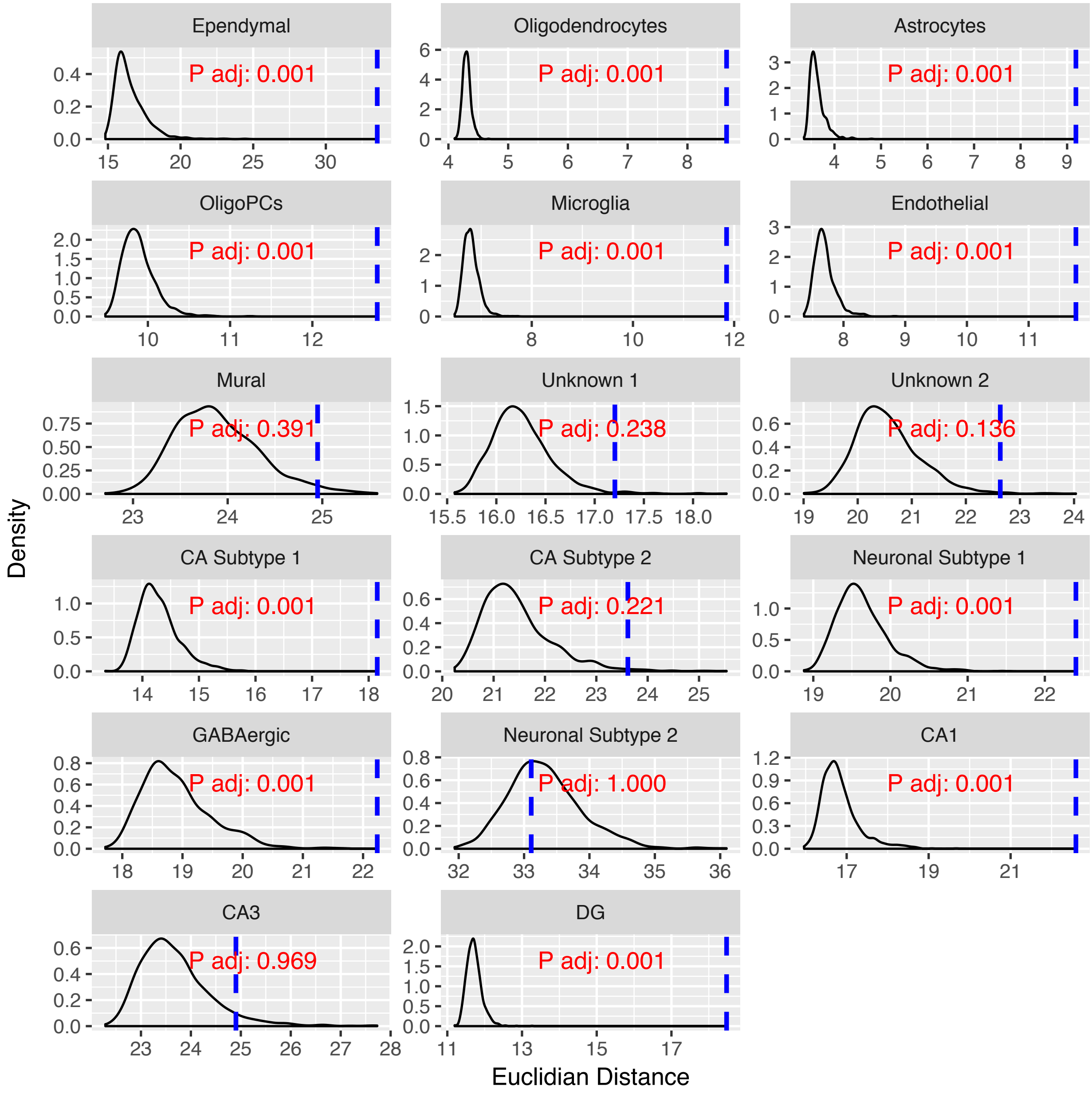

Supplementary Figure 10: Euclidean distance between TBI and Sham single cells. This distance is compared to a null distribution of distances generated by randomly placing single cells from the cell type of interest into two groups the same size as the Sham and TBI groups. The 2D density plot represents the null distribution drawn from each cell type and the blue line indicates the Euclidean distance between Sham and TBI cells from that cell type. The Bonferroni-corrected p-value from 1000 permutations is reported.

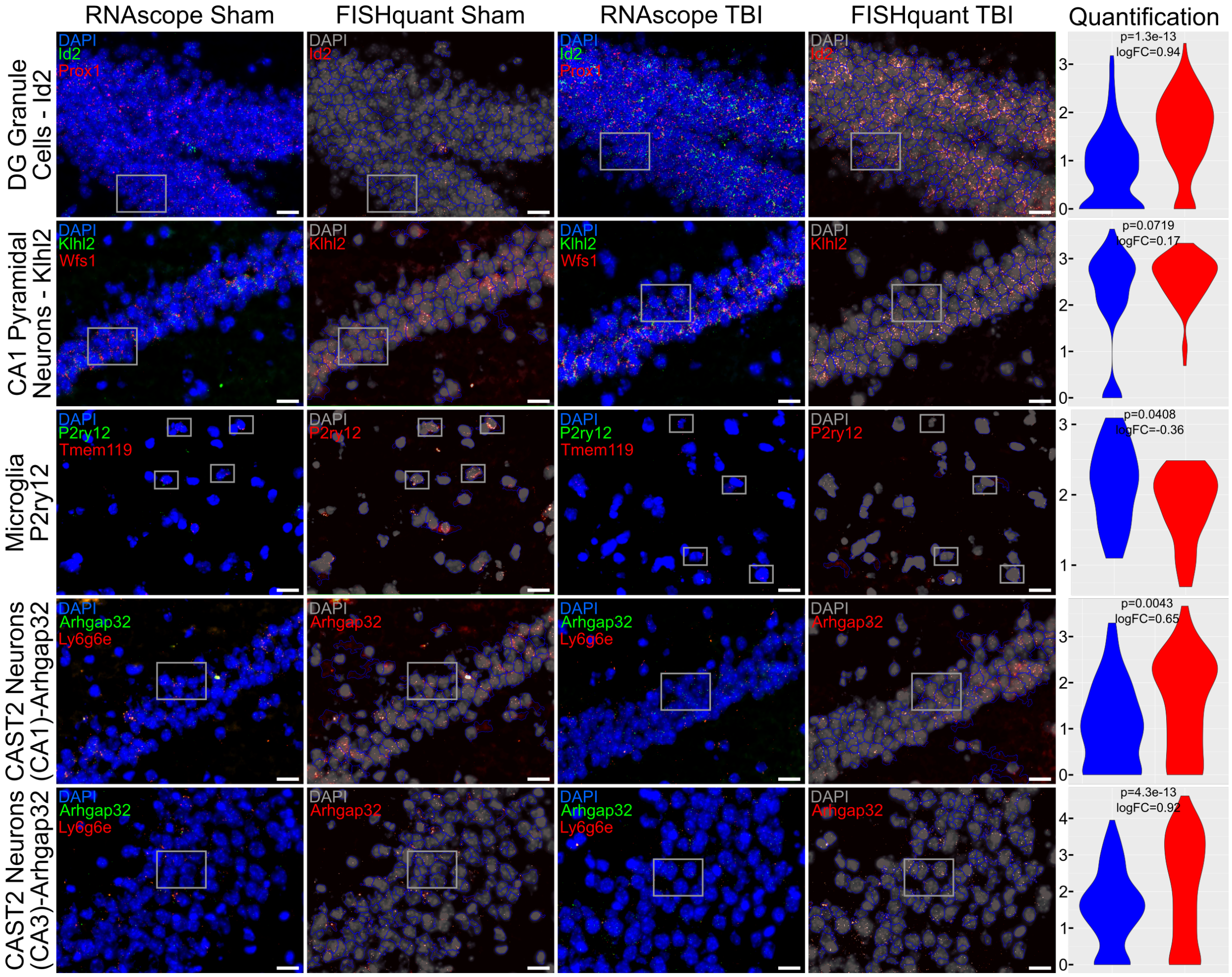

Supplementary Figure 11: Low magnification RNAscope images for cell type specific DEGs. Boxed region corresponds to the magnified region shown in the main text in Fig. 7. Representative fluorescent microphotographs for Sham and TBI showing each DEG of interest along with cell marker genes with DAPI in the background. The RNAscope images for Sham (first column) and TBI (third column) display cell colocalization of each DEG (green) and the corresponding cell marker gene (red). The corresponding FISH-quant images for Sham (second column) and TBI (fourth column) show the automated cell segmentation (blue) overlayed on the 2D maximal projection of the DAPI z-stack images (grey) with the DEG of interest (green in the RNAscope images) indicated in bright dots. The quantification of the cell type specific DEGs is shown in the violin plots (5th column) with Sham in blue and TBI in red. Only cells which meet a certain count threshold for the cell type marker gene are considered the appropriate cell type and used in the DEG quantification (details in Methods). The ln(counts per cell) of the DEGs are shown on the y-axis with the p-value from a bimodal likelihood ratio test and log fold change between TBI and Sham samples indicated. Scale bars are 20um.

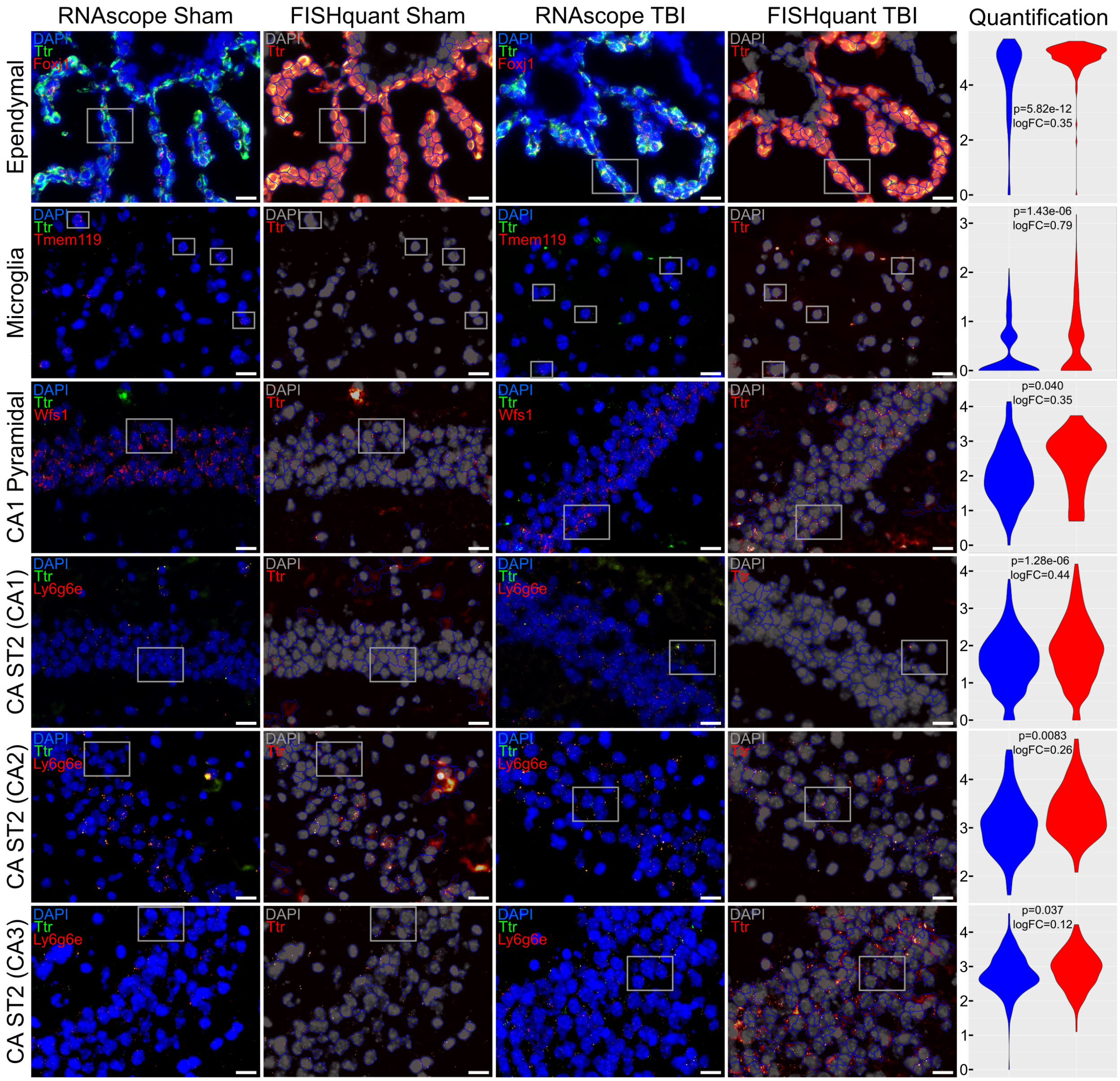

Supplementary Figure 12: Low magnification RNAscope ISH images for Ttr expression. Boxed region corresponds to the magnified region shown in the main text in Fig. 8. Representative fluorescent microphotographs for Sham and TBI showing Ttr along with cell type markers with DAPI in the background. The RNAscope images for Sham (first column) and TBI (third column) display cell colocalization of Ttr (green) and the corresponding cell marker gene (red). The corresponding FISH-quant images for Sham (second column) and TBI (fourth column) show the automated cell segmentation (blue) overlayed on the 2D maximal projection of the DAPI z-stack images (grey) with Ttr (green in the RNAscope images) indicated in bright dots. The quantification of Ttr expression is shown in the violin plots (5th column) with Sham in blue and TBI in red. Only cells which meet a certain count threshold for the cell type marker gene are considered the appropriate cell type and used for Ttr quantification (details in Methods).

The ln(counts per cell) of Ttr is shown on the y-axis with the p-value from a bimodal likelihood ratio test and log fold change between TBI and Sham samples indicated. Scale bars are 20um.

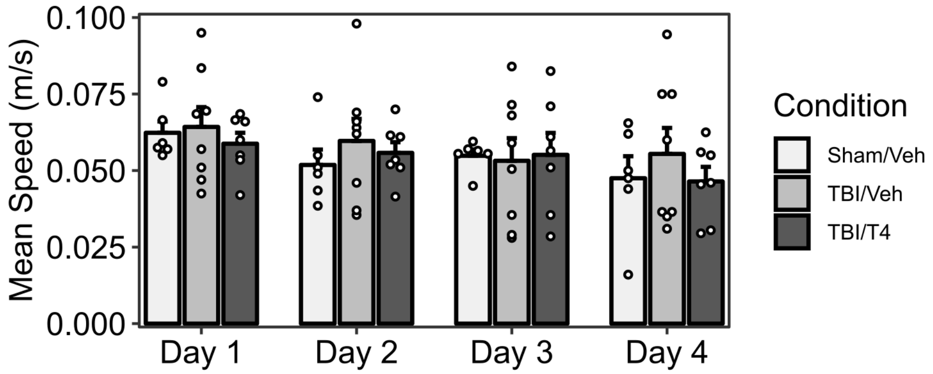

Supplementary Figure 13: Barnes Maze Test animal velocity. The velocity of the Sham, TBI, and TBI/T4 treated mice (Sham/Veh: n=6, TBI/Veh: n=8, TBI/T4: n=7) during the learning phase of the Barnes Maze Test. Error bars are s.e.m.

Supplementary Table 1: Changes in relative cell proportions of individual cell types post-TBI. Counts and fractions of each cell type in the Sham and TBI samples are given along with a Fisher's exact test to see if there is a significant shift in the relative abundance of the cell types due to TBI.

	Ependymal	Neurons	Oligod	Astrocytes	Oligod PCs	Unknown1	Microglia	Unknown2	Mural	Endothelial	Totals
ShamTotal Counts	20	662	785	1187	191	52	249	22	23	223	3414
TBITotal Counts	159	335	561	943	139	61	293	52	24	251	2818
ShamTotal Fraction	0.006	0.194	0.230	0.348	0.056	0.015	0.073	0.006	0.007	0.065	
TBITotal Fraction	0.056	0.119	0.199	0.335	0.049	0.022	0.104	0.018	0.009	0.089	
Fisher's Exact	1.21E-33	4.20E-12	1.84E-02	4.64E-01	2.81E-01	7.00E-02	8.99E-05	1.77E-05	4.64E-01	1.33E-03	

Supplementary Table 2: Significant differentially expressed thyroid hormone receptors induced by TBI in individual cell clusters. P-values and log fold change (in parenthesis) are given.

Gene	Ependymal	Mural	CA Subtype 1	CA1	CA3	DG
Thra	0.013 (-0.67)	0.0038 (0.27)	0.011 (0.55)	6.60E-05 (0.92)	---	0.00050 (-0.52)
Thrb	---	---	---	---	0.0042 (0.91)	---
Thrap3	---	0.032 (0.34)	---	---	---	---
