# Supplementary material for: Single cell molecular alterations reveal target cells and pathways of concussive brain injury
Source: Nat Commun. 2018 Sep 25;9:3894. doi: 10.1038/s41467-018-06222-0 (PMC6156584; doi:10.1038/s41467-018-06222-0)
Supplement: Supplementary file 3 — Description of Additional Supplementary Files [file 41467_2018_6222_MOESM3_ESM.pdf]

Description of Additional Supplementary Files

File Name: Supplementary Data 1
Description: Marker genes for major hippocampal cell clusters.

File Name: Supplementary Data 2
Description: Significant correlations between peptides from source cells and genes in target cells in the cell-cell gene coexpression analysis in Sham and TBI groups.

File Name: Supplementary Data 3
Description: Differentially expressed genes induced by TBI in individual cell clusters.

File Name: Supplementary Data 4
Description: Over-represented biological pathways among DEGs between Sham and TBI for individual cell clusters.

File Name: Supplementary Data 5
Description: Over-represented biological pathways among T4 treatment DEGs (T4 treated TBI vs TBI), TBI DEGs (TBI vs Sham), and TBI DEGs reversed by T4 treatment.
